# Gene expression mapping of conserved GABAergic interneuron markers in developing zebrafish

**DOI:** 10.3389/fnmol.2026.1849805

**Published:** 2026-07-02

**Authors:** Bingyao Zhu, Scott C. Baraban

**Affiliations:** 1 Department of Neurological Surgery, University of California, San Francisco, San Francisco, CA, United States; 2 Developmental and Stem Cell Biology Graduate Program, University of California, San Francisco, San Francisco, CA, United States; 3 Weill Neuroscience Institute, University of California, San Francisco, San Francisco, CA, United States

**Keywords:** GABA, interneuron, progenitor, telencephalon, transcription factor

## Abstract

γ-Aminobutyric acid (GABA)-expressing interneurons play a crucial role in regulating brain activity, and their impairment or loss is linked to various neurological disorders. While mammalian research establishes embryonic ganglionic eminences as the primary GABAergic source, the homologous spatiotemporal developmental pathways and marker profiles in zebrafish remain incompletely characterized. We identified a distinct subpallial region in the telencephalon at 1–2 days post-fertilization (dpf) co-expressing *nkx2.1* and *lhx6a*, transcription factors characteristic of the medial ganglionic eminence (MGE). The caudal ganglionic eminence (CGE) transcription factor *sp8* was expressed in the telencephalon, extending to mesencephalon and rhombencephalon, while *prox1a* expression localized to dorsal diencephalon, preoptic region, mesencephalon, and rhombencephalon. We also observed non-overlapping expression of canonical MGE interneuron markers *sst1.1* (somatostatin) and *pvalb6* (parvalbumin) within telencephalon, diencephalon, and mesencephalon at 3 and 5 dpf. Taken together, these results identify spatiotemporal expression patterns of conserved GE transcription factors and interneuron subtype markers within the developing zebrafish brain, providing an important foundation for using zebrafish models to investigate GABAergic interneuron development and disorders involving inhibitory dysfunction.

## Introduction

Proper brain function, from sensory perception to complex cognition and behavior, relies on precisely coordinated excitatory and inhibitory signaling. This delicate balance is largely mediated by a highly heterogeneous population of inhibitory GABA-expressing interneurons (INs), which regulate excitatory activity, control the timing and synchronization of neural firing and sculpt neuronal circuits (Hu et al., 2011; Klausberger et al., 2003; Pouille and Scanziani, 2001). Loss or dysfunction of GABAergic INs has been implicated in a wide array of neurological pathologies, including epilepsy, Alzheimer’s disease, autism and various other neuropsychiatric conditions (Baraban et al., 2009; Gogolla et al., 2009; Verret et al., 2012). To understand how inhibitory circuits function, it is essential to first establish the spatiotemporal origins of their cellular diversity. Because GABAergic INs encompass diverse subtypes with unique molecular, morphological, and physiological properties, elucidating these developmental trajectories remains a central challenge in neuroscience.

In mammalian brain, GABAergic INs primarily originate in a transient embryonic structure known as the ganglionic eminence (GE), located within a developing telencephalon (Anderson et al., 1997). The GE is subdivided into three sub-regions: medial (MGE), lateral (LGE) and caudal (CGE), each defined by distinct molecular markers and anatomical locations. MGE, which appears first at the telencephalic-diencephalic junction, expresses canonical markers *Nkx2.1* and *Lhx6* (Du et al., 2008; Liodis et al., 2007; Sussel et al., 1999). It predominately generates parvalbumin (PV)- and somatostatin (SST)-expressing INs (Xu et al., 2004). In mammals, almost 70% of cortical interneurons originate in the MGE. LGE lies ventro-lateral to MGE and contributes mainly to olfactory bulb INs (de Carlos et al., 1996; Stenman et al., 2003; Wichterle et al., 1999; Yun et al., 2001). CGE, positioned posterior to the sulcus separating MGE and LGE, lacks a definitive physical boundary, leading to ongoing debate about its precise anatomical definition and transcription factor profile (Miyoshi et al., 2010; Nery et al., 2002). Currently, *Coup-tfII*, *Sp8* and *Prox1*, whose expression is enriched in CGE, serve as its primary molecular markers (Kanatani et al., 2008; Miyoshi et al., 2015; Wei et al., 2019). CGE mainly produces late-born INs expressing calretinin, calbindin, vasoactive intestinal peptide and reelin destined for cortex, hippocampus and amygdala locations (Lee et al., 2010; Miyoshi et al., 2010; Touzot et al., 2016). A small subset of neurogliaform cortical interneurons originating in preoptic area (PoA) also express the homeobox-encoding *Prox1* gene (Niquille et al., 2018; Rubin and Kessaris, 2013).

Although this developmental framework is well characterized in mammals, whether a comparable tripartite organization exists in teleosts such as zebrafish (*Danio rerio*) is not as well documented (Mueller et al., 2006; Mueller et al., 2008). This represents a critical deficiency as zebrafish have emerged as a powerful model organism in translational neuroscience for functional analysis of human gene mutations, high-throughput drug screening and brain-wide imaging of network function (Ahrens et al., 2013; Baraban et al., 2005; Griffin et al., 2021; Howe et al., 2013; Moog and Baraban, 2022; Whyte-Fagundes et al., 2024). Zebrafish models of interneuron-related brain disorders, for example epilepsy associated with an interneuron-specific sodium channel subunit mutation (*SCN1A*), have already facilitated important advances in understanding epileptogenesis and the discovery of novel antiseizure medications (Moog and Baraban, 2022). Research on neurotransmitter content and neuronal structure of GABAergic neurons in larval zebrafish has mostly focused on the spinal cord (Bernhardt et al., 1990; Higashijima et al., 2004; Pallucchi et al., 2024; Satou et al., 2012; Wyart et al., 2009) with a more limited literature on larval forebrain or midbrain structures (Mueller et al., 2008; Ramaswamy et al., 2020; Robles et al., 2011).

To address this gap, we employed whole-mount multiplexed Hybridization Chain Reaction (HCR) staining to evaluate expression of conserved GE specific transcription factors and molecular markers of mature INs. The small size, rapid *ex vivo* development, and optical transparency of larval zebrafish facilitate whole-mount HCR, allowing detailed analysis of gene expression across the entire nervous system (Choi et al., 2018; Schulte et al., 2024). Here, we mapped expression of gene transcripts for *nkx2.1*, *lhx6a* (MGE), *coup-tfII*, *sp8* (CGE), *prox1a* (CGE & LGE), *dlx5a/6a* (MGE, LGE & CGE) from zebrafish telencephalon, diencephalon, mesencephalon and rhombencephalon sub-regions at 1/2-, 3- and 5–6 days post-fertilization (dpf). Our results indicate that co-expression of *nkx2.1* and *lhx6a* distinctly identifies an MGE region within the developing telencephalon (subpallium); *sp8*, *prox1a* and *coup-tfII* were not expressed in this subpallial region. In addition, we found non-overlapping expression of mature interneuron markers (*sst1.1*, *pvalb6*, *vip*, *calb2a/2b*). This work serves not only as a developmental reference for cross-species comparisons, but also as a foundational resource for dissecting inhibitory interneuron maturation, diversity and function in future studies.

## Materials and methods

### Zebrafish husbandry

Zebrafish (*Danio rerio*) were maintained in a temperature-controlled facility on a 14 h:10 h light/dark cycle under standard conditions (28–30 °C, pH: 7.5–8, conductivity: 690–740 mS/cm). For spawning, adult pairs were separated overnight by a divider, which was removed the following morning for synchronized egg collection. Embryos and larvae were reared in E3 medium (0.03% Instant Ocean in RO-distilled water) at 28.5 °C up until 6 dpf. All experimental procedures were performed in accordance with Institutional Animal Care and Use guidelines. For all experiments larvae were randomly selected from clutches containing 100–200 healthy embryos.

### Multiplexed hybridization chain reaction (HCR)

Spatial expression of interneuron markers was mapped using 3rd generation *in situ* hybridization chain reaction (HCR, Choi et al., 2018). DNA antisense oligonucleotide probe sets (12–20 pairs per gene) were designed with split-initiator sequences for auto background suppression and synthesized by Integrated DNA Technologies (IDT, Supplementary Table 1). Staining was performed according to established protocols (Lovett-Barron et al., 2020). Briefly, Larvae (1–6 dpf) were fixed in 4% paraformaldehyde (PFA) overnight at 4 °C, dehydrated in 100% methanol for 20 min at −20 °C and sequentially rehydrated in a methanol/SSCT gradient. Samples were incubated in hybridization buffer (4 nM probes, 10% dextran sulfate and 10% formamide in 2 × SSCT) overnight at 37 °C. Following washes (30% formamide in 2 × SSCT), signal amplification was performed using B1–5 hairpins (Molecular Instrument, 240 nM) conjugated to Alexa Fluor 488, 546 or 647. Hairpins were snap-cooled (95 °C for 1 min then 20 min at RT) prior to overnight incubation in the dark in amplification buffer (10% dextran sulfate in 5 × SSCT). Samples were stored in 1 × PBST at 4 °C until imaging.

### Confocal microscopy

HCR-stained larvae were embedded in 1% low-melting agarose on 35 mm glass-bottom FluoroDish (WPI). Imaging was performed on a Nikon confocal microscope using 10×/0.45 (Plan Apo) or 20×/0.70 (Plan FL) air objectives. XY resolutions were set at 512 or 1,024 pixels, with z-step intervals (voxel depth) of either 2 μm or 8 μm depending on the required volumetric detail. To ensure consistent signal across samples, laser power and detector gain were calibrated to the specific marker and stage. Due to light attenuation and objective working distance, imaging depth was focused on the first 80 μm of the brain to maintain optimal signal-to-noise ratio.

### Machine learning denoising and image processing

To enhance signal clarity, 3D stacks were denoised using the Noise2Void (N2V) self-supervised deep learning framework (Krull et al., 2018). To minimize grid-like artifacts associated with standard up sampling, we modified the U-Net architecture by increasing the convolution kernel from 3 × 3 to 5 × 5 throughout the network. The model was trained for 100 epochs on a representative dataset of images (64 × 64 pixel patches, batch size 128) utilizing a blind spot masking scheme to predict pixel intensities from local neighborhoods. Optimization was performed via Mean Squared Error (MSE) loss. The trained model was applied slice-by-slice across all channels. All computational pipelines were implemented in Python (v3.10) using n2v (v0.3.2) csbdeep and TensorFlow (v2.12) backend.

## Results

### Transcriptomic visualization of conserved interneuron signatures in the zebrafish brain

**Figure 1:**
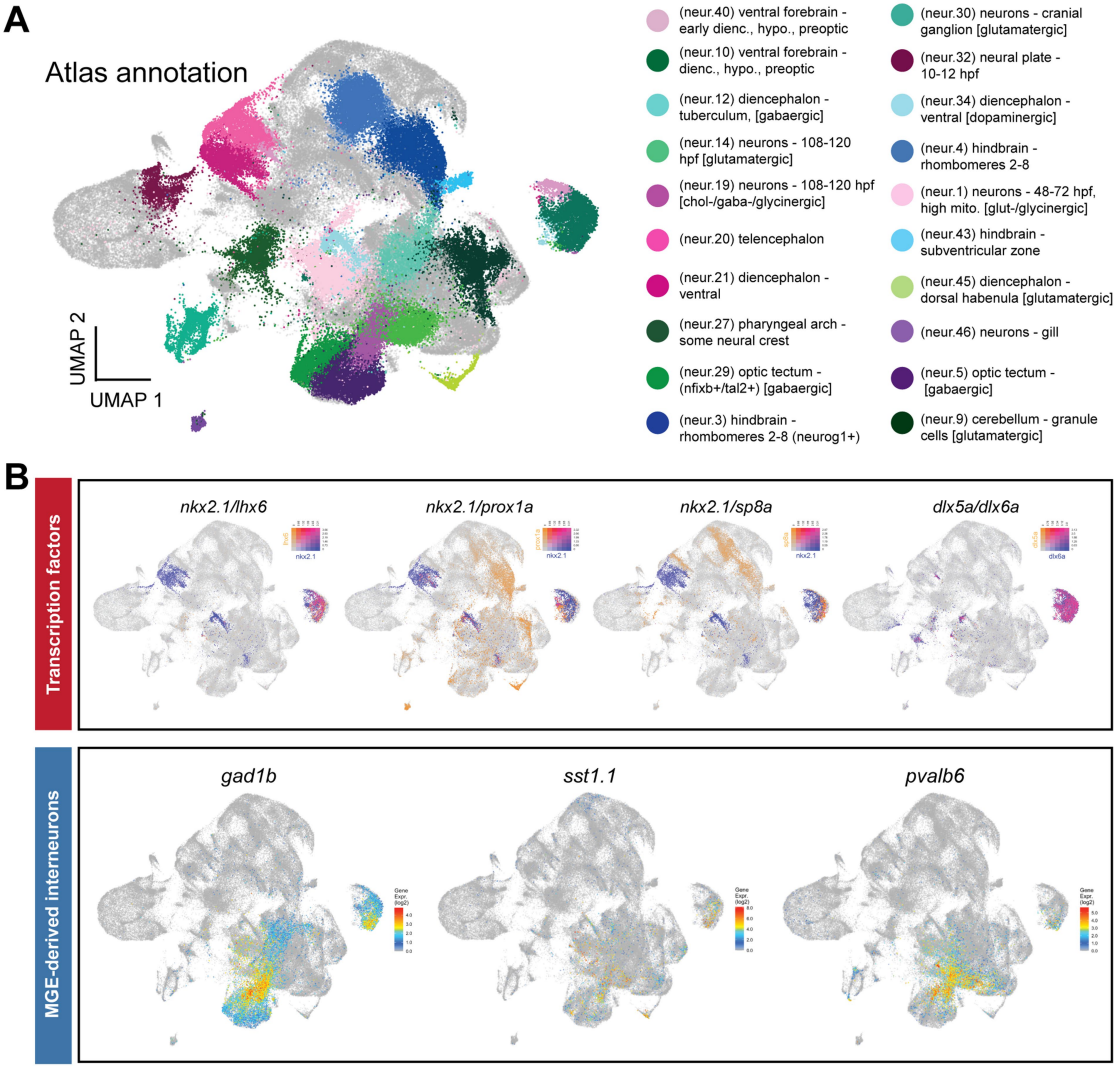
Transcriptomic visualization of conserved interneuron signatures in the zebrafish brain. **(A)** Annotated UMAP projection of the DanioCell single-cell RNA sequencing dataset. Visualizing major neuronal clusters. Colors correspond to distinct cell identities with key forebrain clusters highlighted. **(B)** Feature plots showing the expression of conserved progenitor and interneuron marker genes. Top: expression of transcription factors defines distinct progenitor domains. *nkx2.1* and *lhx6a* are co-expressed in ventral forebrain clusters, while *prox1a* and *sp8* mark separate populations. Bottom: distribution of the pan-GABAergic marker *gad1b* and canonical MGE subtype markers. Note that *sst1.1* and *pvalb6* are resolved into non-overlapping neuronal populations within the broad GABAergic clusters.

To begin to understand the developmental origins of interneurons, we first visualized gene expression patterns using a publicly available single-cell RNA sequencing (scRNA-seq) database spanning 3 h post-fertilization to 5 dpf (Sur et al., 2023) (Figure 1A). We hypothesized that immature zebrafish brain contains discrete progenitor domains analogous to mammalian MGE, LGE and CGE, each defined by a conserved combination of transcription factors (TFs). In this proposed model, the MGE-like domain is characterized by *nkx2.1* and *lhx6a* expression, while LGE and CGE-like territories are associated with *dlx5a/6a*, *prox1a*, *sp8* and *coup-tfII*.

Visualizations retrieved from the DanioCell Uniformed Manifold Approximation and Project (UMAP) dataset confirmed molecular signatures that align with this proposed tripartite organization. Specifically, canonical MGE markers *nkx2.1* and *lhx6a* were highly enriched and co-expressed within a specific cluster of ventral forebrain neurons (cluster [neur.10, neur.40], Figure 1B). Clusters representing ventral diencephalon [neur. 21] and GABAergic neurons [neur.12 and neur.19] were only enriched for *nkx2.1*. Co-expression of *dlx5a* and *dlx6a*, transcription factors marking most GABAergic interneurons in zebrafish (Robles et al., 2011; Yu et al., 2011), was also observed in this same ventral forebrain cluster. However, markers for genes transcription factors typically associated with LGE/CGE, e.g., *prox1a* and *sp8* in hindbrain (clusters [neur.3 and neur.4]) or telencephalon (cluster [neur.20]) did not co-localize with *nkx2.1* (Figure 1B). Furthermore, major MGE GABAergic subtypes, *sst1.1* and *pvalb6*, were identified in neuronal population clusters enriched for GABAergic neurons [neur.5, neur.19 and neur.12] of the optic tectum and diencephalon (tuberculum) (Figure 1B).

### Anatomical characterization of a ventrally restricted MGE-like domain at 1–2 dpf

**Figure 2:**
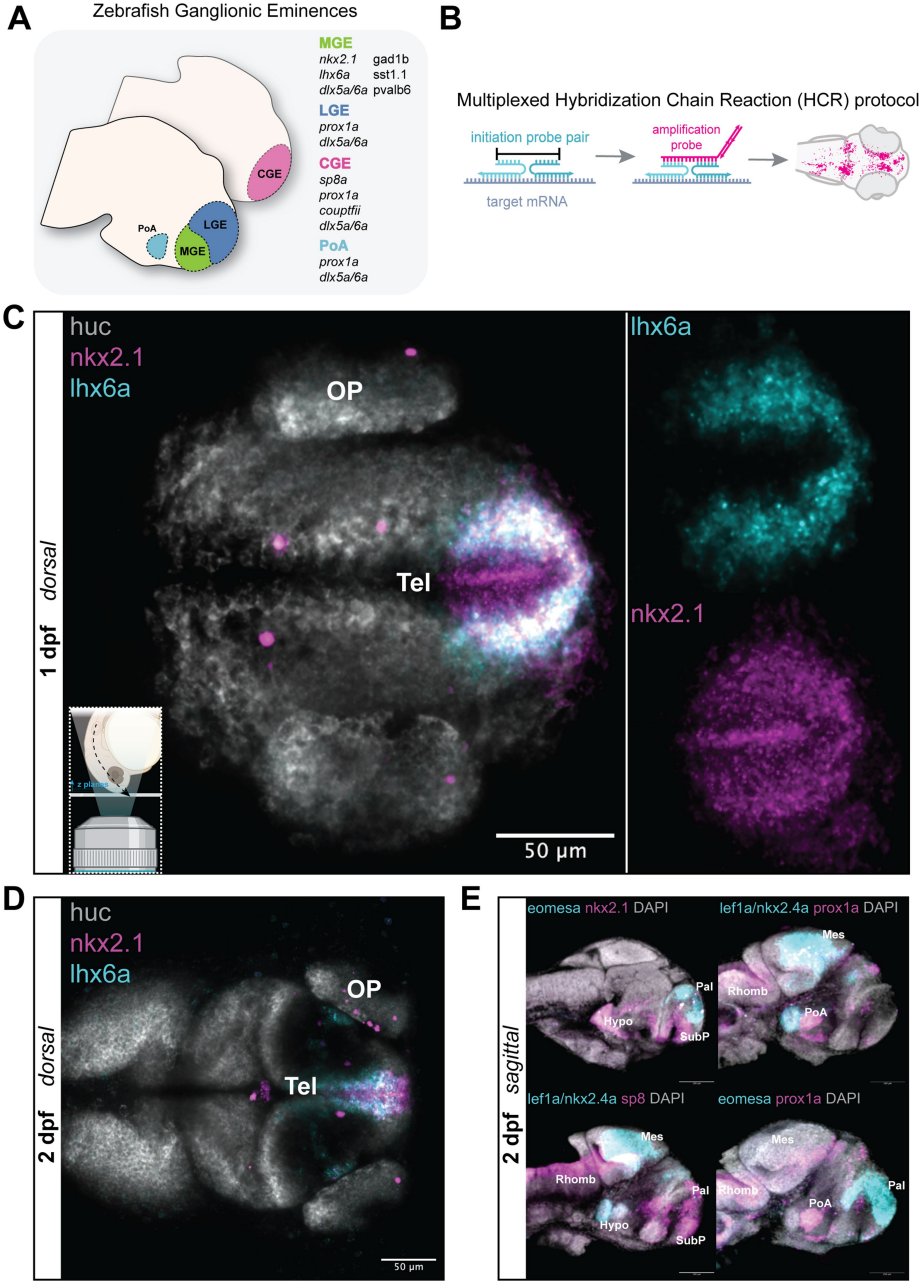
Anatomical characterization of the subpallial MGE-like domain at 1–2 dpf. **(A)** Schematic representation of the hypothesized zebrafish GEs, listing the combination of markers used for identification. **(B)** Schematic workflow of the whole-mount multiplexed HCR protocol. **(C, D)** *maximum*/average intensity projection of dorsal forebrain views at 1 dpf (**C**, *n* = 3) and 2 dpf (**D**, *n* = 5). HCR staining for nkx2.1 (magenta) and lhx6a (cyan) reveals a stable, co-expressed domain in the subpallium. Huc (gray, average intensity projection) serves as a pan-neuronal counterstain. The inset in **(C)** indicates the imaging plane. **(E)** Sagittal optical sections at 2 dpf visualizing the boundaries of the MGE-like domain against adjacent territories. nkx2.1 expression is restricted to the subpallium and hypothalamus. In contrast, prox1a and sp8 were expressed throughout the diencephalon, mesencephalon and rhombencephalon, with sp8 extending rostrally into the telencephalon. Eomesa (pallium), lef1a (optic tectum, hypothalamus) and nkx2.4a (hypothalamus, all cyan) serve as regional anatomical markers (*n* = 4–8 larvae per condition). Hypo, Hypothalamus; Mes, Mesencephalon; OP, olfactory placode; Pal, Pallium; PoA, Preoptic area; Rhomb, Rhombencephalon; SubP, Subpallium; Tel, Telencephalon.

**Figure 3:**
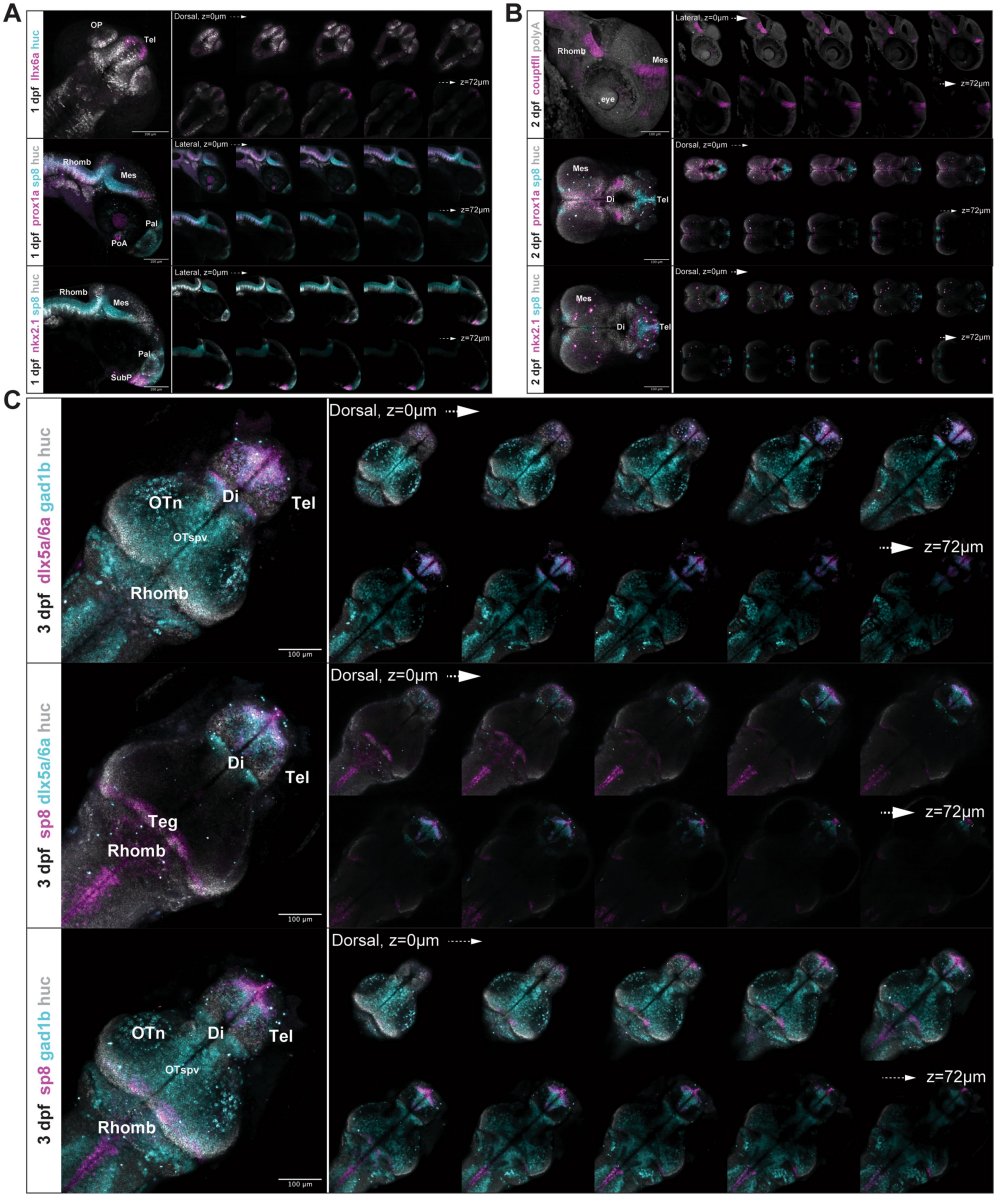
Spatial segregation of progenitor domains and establishment of GABAergic identity. **(A–C)** Confocal analysis of interneuron marker expression. For all representative stacked images (left panel), the huc/polyA counterstain (gray) is displayed as an average intensity projection to visualize anatomical architecture, while gene markers are maximum intensity projections. Corresponding serial z-stack montages (right panel) display optical sections separated by 8 μm intervals. **(A,B)** GE marker gene expression at 1 dpf **(A)** and 2 dpf **(B)**. multiplexed HCR for prox1a, sp8 and nkx2.1 shows largely non-overlapping territories. **(C)** GABAergic establishment at 3 dpf. dlx5a/6a (magenta) and gad1b (cyan) have a high degree of spatial correlation, confirming the subpallium as a primary GABAergic progenitor zone. (*n* = 4–6 larvae per condition). Tel, Telencephalon; Di, Diencephalon; Mes, Mesencephalon; Rhomb, Rhombencephalon; Pal, Pallium; SubP, Subpallium; OTn, Optic Tectum; OTspv, Optic Tectum Stratum Periventriculare.

While transcriptomic profiles support a conserved molecular framework, they lack the anatomical resolution required to define physical boundaries of GE progenitor zones (Figure 2A). To resolve the spatiotemporal expression of MGE progenitors, we performed multiplexed HCR staining for *nkx2.1* and *lhx6a* starting at 1 and 2 dpf (Figure 2B). Zebrafish brain regions were identified using DAPI blue, fluorescent staining or pan-neuronal markers such as *HuC* or *PolyA*. At the onset of specification (1 dpf), *nkx2.1* expression was broadly distributed across subpallium (Figure 2C; Supplementary Figure 2-1). We observed that *lhx6a* expression largely overlapped with the *nkx2.1* domain but was confined to a more restricted subset of the *nkx2.1* territory (Figures 2C, 3A). By 2 dpf, co-expression of *nkx2.1* and *lhx6a* remained highly stable (Figure 2D), consistent with assigning an MGE-like identity to this structure during early forebrain patterning (Figure 2A). However, the spatial distribution of *nkx2.1* was notably expansive, extending beyond telencephalic boundaries into hypothalamus (Figure 2E). This pattern indicates that while *nkx2.1* is a primary driver of subpallial interneuron identity, it may also serve as a broader marker for ventral forebrain specification in zebrafish.

To further delineate the MGE-like region as a distinct anatomical entity, we next characterized expression of *sp8* and *prox1a* as markers for adjacent non-MGE domains. At 1 dpf, *sp8* was localized to telencephalon, specifically pallium and rostral subpallium and extended through the posterior half of mesencephalon, across the midbrain-hindbrain boundary (MHB) into the rhombic region, a distribution that remained stable at 2 dpf (Figures 2E, 3A,B). In parallel, *prox1a* at 1 dpf was localized to dorsal diencephalon, preoptic region, anterior half of mesencephalon and rhombic regions (Figure 3A). By 2 dpf, the *prox1a* domain expanded into diencephalic habenula (Figures 2E, 3B). Notably, *sp8* and *prox1a* occupied largely non-overlapping territories, with convergence observed only within the rhombic region. Most importantly, *sp8* and *prox1a* remained mutually exclusive with the *nkx2.1* expression domain (Figures 3A,B). Furthermore, *couptfII* expression was not observed in pallium but was restricted to small patches in anterior mesencephalon and rhombencephalon at 2 dpf (Figure 3B). In aggregate this segregation further supports a conclusion that *nkx2.1*/*lhx6a*-enriched territory constitutes a molecularly and spatially defined MGE-like structure. Collectively, these findings provide compelling evidence that zebrafish ventral forebrain possesses a conserved developmental origin for inhibitory interneurons that is spatially distinct from other subpallial progenitor pools (Figure 2A).

### Distribution of GABAergic markers within telencephalon at 3 dpf

Building on establishment of early progenitor territories, we next investigated the emergence of GABAergic interneuron identity at 3 dpf. To define the broader subpallial landscape, we first examined the expression of *dlx5a/6a*. Beyond their use as pan-subpallial markers, *dlx5a/6a* serve as critical determinants of GABAergic fate, influencing the specification of various inhibitory lineages (MacDonald et al., 2010; Yu et al., 2011; Zerucha et al., 2000). In telencephalon (forebrain), *dlx5a/6a* expression was prominent in subpallium, a pattern that persisted up to 5 dpf (Supplementary Figure 3-1). Dual HCR staining demonstrated a relatively high degree of spatial correlation between *dlx5a/6a* and the pan-GABAergic marker *gad1b*, confirming that *dlx5a/6a* expression in subpallium marks the primary site of inhibitory interneuron specification (Figure 3C). In contrast, *sp8* expression occupied a highly restricted niche at the rostral tip of telencephalon, tegmentum in dorsal mesencephalon and down the midline in rhombencephalon (Figure 3C). Within these regions, *sp8* exhibited limited spatial overlap with both *dlx5a/6a* and *gad1b*, with co-distribution confined primarily to a posterior edge of the *sp8* domain (Figure 3C). Collectively, this mapping defines zebrafish subpallium as a *dlx5a/6a* expressing GABAergic progenitor zone, physically segregated from the *sp8* domain at the rostral pole.

### Progressive spatial distribution of mature interneuron subtypes between 3 and 5 dpf

**Figure 4:**
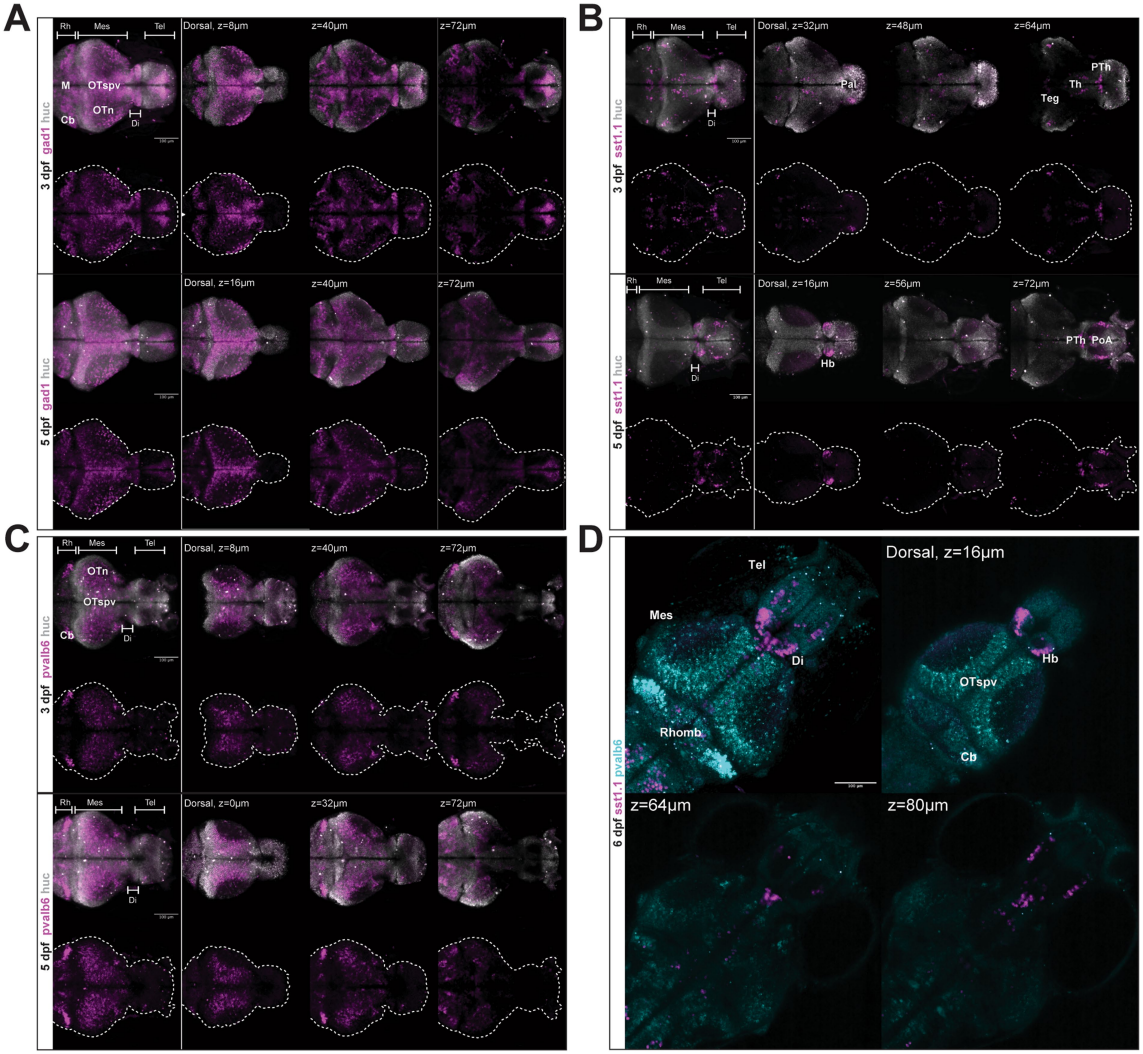
Progressive spatial distribution of terminal interneuron subtypes between 3 and 5 dpf. **(A–D)** Confocal analysis of terminal interneuron subtypes. For all images, the huc counterstain (gray) is displayed as an average intensity projection to visualize anatomical architecture, while gene markers are maximum intensity projections. **(A)** Expression of the pan-GABAergic marker gad1b (magenta) at 3 dpf (top) and 5 dpf (bottom). Representative stacked images (left) and z-sections (right) revealed a robust inhibitory framework distributed throughout all major subdivisions of the zebrafish brain. **(B)** Spatial organization of the MGE-derived interneuron subtype marker sst1.1 (magenta). Expression is scattered within the forebrain, hypothalamus and rhombencephalon. **(C)** Distribution of the MGE-derived interneuron marker pvalb6 (magenta). In contrast to sst1.1, pvalb6 expression is sparse in the forebrain, but highly enriched in the optic tectum and cerebellum. **(D)** Dual multiplexed HCR at 6 dpf showing sst1.1 (magenta) and pvalb6 (cyan). High-resolution optical sections demonstrate that these markers occupy discrete territories with no observable co-expression, confirming they delineate separate neuronal populations. (*n* = 4–7 larvae per condition). Tel, Telencephalon; Di, Diencephalon; Mes, Mesencephalon; Rhomb, Rhombencephalon; M, Medulla Oblongata; Cb, Cerebellum; Pal, Pallium; SubP, Subpallium; OTn, Optic Tectum; OTspv, Optic Tectum Stratum Periventriculare.

To evaluate distribution of differentiated interneuron populations, we performed multiplexed HCR for mature interneuron subtype markers: *sst1.1* and *pvalb6* (MGE), *calb2a/2b* and *vip* (CGE) along with a pan-GABAergic marker *gad1b* between 3 and 5 dpf (Figure 4; Supplementary Figure 4-1). At 3 dpf, *gad1b* expression was robustly distributed throughout zebrafish brain (Figures 3C, 4A). Within this broad inhibitory framework, expression of canonical markers for MGE interneuron subtypes revealed highly regionalized patterns. For example, *pvalb6* expression was most prominent within optic tectum and cerebellum, whereas its presence in telencephalon (forebrain) remained sparse (Figure 4C). In contrast to the broad anterior-to-posterior distribution of *pvalb6*, *sst1.1* expression was mostly limited to ventral forebrain. Scattered labeling was observed in forebrain structures including habenula, thalamus, hypothalamus and hindbrain (Figure 4B). Examination of CGE interneuron markers showed a similarly distinct regional expression. Notably, *calb2a/2b* showed robust expression throughout optic tectum, pretectum and cerebellum (Supplementary Figure 4–1). The distribution of *vip*, however, is sparse and substantially more restricted. It appeared only as rare, solitary cells embedded within thalamus and hypothalamus (Supplementary Figure 5-1). By 5 dpf, spatial organization of these mature interneuron markers largely remained consistent with patterns observed at 3 dpf (Figures 4; Supplementary Figures 3-1, 4-1) consistent with the early functional capacity of larval zebrafish.

To characterize the spatial arrangement of MGE-derived interneuron populations relative to one another, we also examined dual stained zebrafish at 6 dpf. We found that *sst1.1* and *pvalb6* occupied strictly discrete territories, suggesting the two markers of MGE-derived interneurons occupy non-overlapping regions (Figure 4D).

### Construction of a spatiotemporal expression matrix of interneuron specification

**Figure 5:**
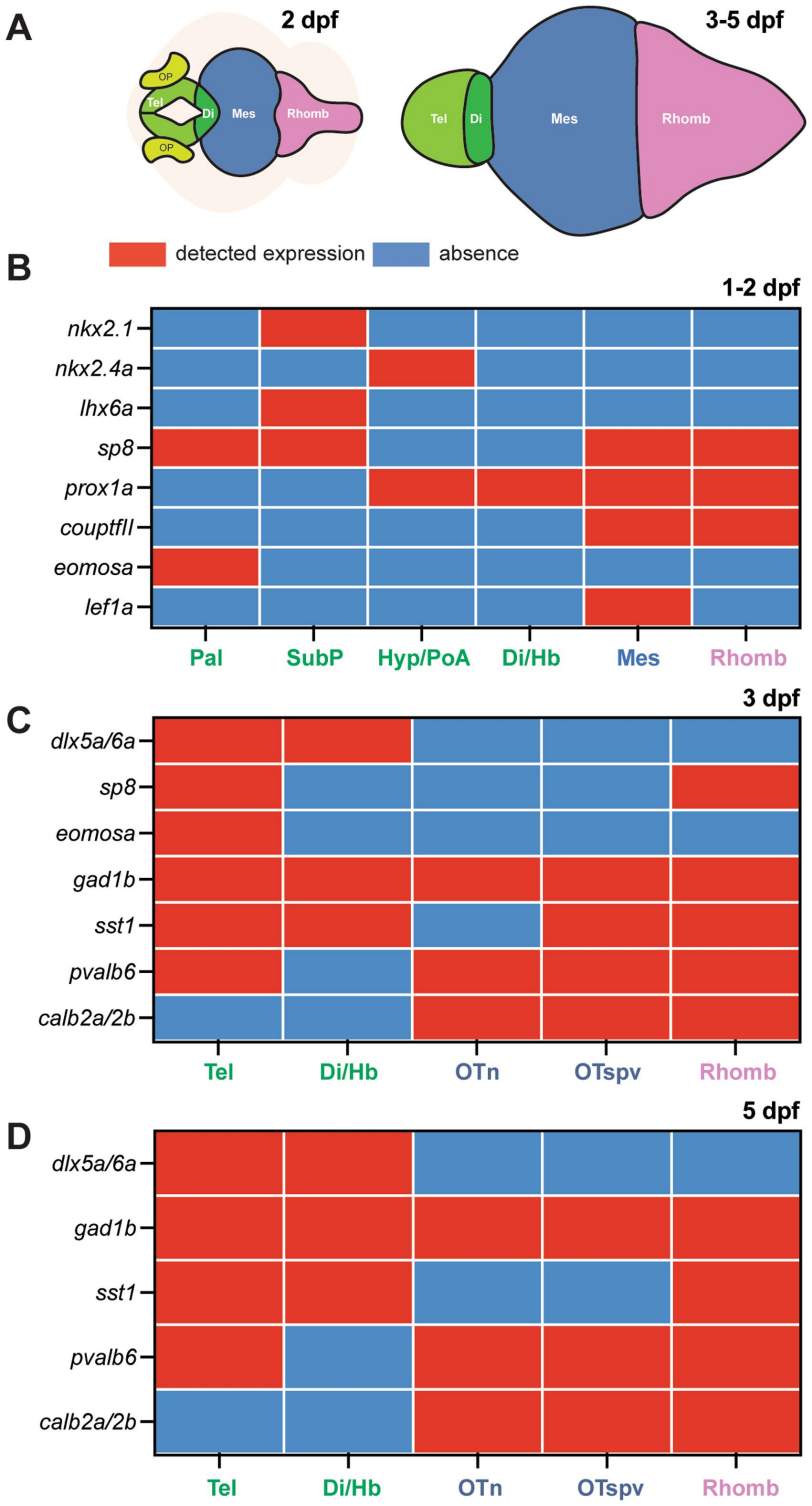
Spatiotemporal expression matrix of interneuron markers in the developing zebrafish brain. **(A)** Schematic representation of the developing zebrafish brain at 2 dpf (left) and 3–5 dpf (right). Illustrating major anatomical subdivisions. Regions are color-coded to correspond with the heatmap columns. **(B)** Heatmap summarizing the regional expression of progenitor transcription factors at 1–2 dpf. Rows represent specific genes, and columns represent anatomical subdivisions. Red indicates detected expression, blue indicates absence. **(C,D)** Expression profiles of pan-GABAergic (dlx5a/6a, gad1b) and interneuron subtype markers (sst1.1, pvalb6, calb2a/2b) at 3 dpf **(C)** and 5 dpf **(D)**. Tel, Telencephalon; Di, Diencephalon; Mes, Mesencephalon; Rhomb, Rhombencephalon; OP, olfactory placode; Pal, Pallium; SubP, Subpallium; Hyp, Hypothalamus; PoA, Preoptic Area; Hb, Habenula.

To consolidate the complex expression patterns observed across early larval development, we generated binary heatmaps summarizing regional distribution of major GE and interneuron markers from 1 to 5 dpf (Figure 5). Due to differences in HCR staining intensity across *in vivo* preparations quantification was not performed. As a descriptive summary, heatmaps using a simple binary scoring system: ‘no gene expression’ (0, blue) or ‘gene expression observed’ (1, red) within each brain region were generated. This approach provides a global summary of where each marker is expressed within established anatomical subdivisions of the developing zebrafish brain. At the early progenitor stage (1–2 dpf), we observed distinct regional preferences in telencephalon (forebrain). Notably, canonical MGE markers *nkx2.1* and *lhx6a* were detected exclusively in subpallium, contrasting CGE- and LGE-associated markers *sp8*, *prox1a* and *couptfII* with broader expression patterns that include mesencephalon and rhombencephalon. As development progresses to 3 and 5 dpf, distribution of interneuron specific markers reflects the expected migration and maturation of GABAergic interneurons. While *gad1b* was detected across all major brain divisions, upstream selectors like *dlx5a/6a* remained strictly confined to forebrain regions (Figures 5C,D). Among mature interneuron subtype markers, *calb2a/2b* was predominately found in mid- and hindbrain territories, while being absent from telencephalon. Whereas *sst1.1* resided within ventral forebrain and hindbrain and *pvalb6* exhibited broad expression across the anterior–posterior axis. Collectively, this summary illustrates the dynamic spatial landscape of GABAergic marker expression throughout early zebrafish development.

## Discussion

Medial, lateral, or caudal ganglionic eminence region-specific origins, routes, and destinations of zebrafish interneurons likely exist, as shown in mammals (Casalia et al., 2021; Hansen et al., 2013; Nery et al., 2002; Xu et al., 2004). However, an integrative map of GE regions in developing zebrafish is unavailable. Migration studies by Mueller et al. using the *dlx5a/dlx6a*-GFP transgenic line suggested a dorsal telencephalic site of GABAergic progenitor cells emerging at 28 hpf (Mueller et al., 2006), but this was not confirmed using markers that define ganglionic eminence sub-regions. Here we used a multiplexed HCR approach focusing primarily on MGE-specific transcription factors to resolve, for the first time, the precise boundaries of this progenitor territory. Our study shows that the molecular logic of mammalian MGE in the context of interneuron development is conserved in teleost.

Dorsal views of whole-mount HCR staining of larvae at 1–2 dpf showed largely overlapping expression of *nkx2.1* and *lhx6a* in a region corresponding to dorsal telencephalon (Figure 2). Previous anatomical study utilizing chromogenic *in situ* hybridization (Manoli and Driever, 2014) demonstrated robust *nkx2.1* expression localized to basal telencephalon and hypothalamus areas during early larval development. Our multiplexed HCR approach directly builds upon these foundational observations. Imaging *nkx2.1* and *eomesa* (*Tbr2*, a pallium-specific marker) co-expression in 2 dpf larvae from a sagittal perspective confirmed a distinct sub-division of sub-pallium where *nkx2.1* is expressed (Figure 2E). Furthermore, sagittal images further showed a segregation of a putative MGE domain from adjacent territories with mostly non-overlapping *nkx2.1* expression alongside *sp8* and *prox1a*, which further extend beyond telencephalon into mesencephalic and hypothalamus/PoA compartments (Figure 2E). This compartmentalization mirrors the spatial organization of mammalian GEs, where distinct progenitor pools are physically separated to facilitate independent developmental programs (Nery et al., 2002; Sussel et al., 1999; Yun et al., 2001).

Genes for the glutamate decarboxylase enzymes that convert glutamate into GABA (e.g., *gad1a* and *gad1b*) are commonly used as pan-neuronal GABAergic interneuron markers. Immunohistochemistry studies in several species have shown largely overlapping expression of *gad1a* and *gad1b* in the developing brain, including studies of the zebrafish brain at 4 dpf (Filippi et al., 2014). Previous zebrafish work also showed that *gad* expressing cells can be seen in tissue slices containing dorsal subpallium extending toward pallium (Mueller et al., 2008) at 2–3 dpf suggesting the equivalent of a zebrafish MGE. In these studies, *lhx6a* expression in subpallium was also described. Consistent with these findings, we observed prominent *nkx2.1* and *lhx6a* co-expression at 1–2 dpf corresponding to the subpallium in whole-mount studies (Figure 2C). As early as 3 dpf, *gad1b* expression extends into mesencephalon (optic tectum) and the anterior portion of rhombencephalon (cerebellum) suggesting migration of these neurons in an anterior-to-posterior direction (Figure 4A). At 5 dpf, as shown previously, *gad1b* expression occupies all regions of the larval brain (Figure 4A). These patterns of expression are largely similar to those seen in the adult zebrafish (Mueller and Guo, 2009) suggesting that a mature functional GABAergic network is established very early in development. That synchronized seizure activity can be elicited with a GABA receptor antagonist (PTZ) as early as 5 dpf (Baraban et al., 2005) is consistent with this interpretation.

At 3 dpf, we observed the emergence of canonical MGE-derived interneuron subtype markers like *sst1.1* and *pvalb6* (Figures 4B,C). A scattered distribution of *sst1* expression was seen along the midline in regions corresponding to pre-thalamus, thalamus and tegmentum with more prominent expression at 5–6 dpf primarily in diencephalon (habenula) (Figure 4B). Expression of pre-prosomatostatin (*PPS1*) from 55 hpf to 5 dpf in diencephalon was initially reported by Devos et al. (2002). A Gal4: UAS *sst3* reporter line developed by the Baier laboratory reported scattered single cells in hindbrain at 6 dpf (Forster et al., 2017). In contrast, more diffuse expression of *pvalb6* was seen at 3 dpf in mesencephalon extending to rhombencephalon (cerebellum) at 5 dpf (Figure 4C). Immunohistochemistry studies using an antibody recognizing parvalbumin, identified Parv-positive cell bodies in optic tectum stratum periventriculare (OTspv) region lying close to the neuropil in sagittal views at 5 dpf (Nevin et al., 2008). Dorsal views of whole-mount *in situ* hybridization using a probe for *pvalb6* confirmed these findings and also showed more scattered expression in neuropil as well as prominent expression in cerebellum at 5–6 dpf. Consistent with mammals, non-overlapping expression of *sst1* and *pvalb6* was most evident at 6 dpf in the zebrafish brain.

While this study establishes an integrative molecular reference for an MGE-like progenitor zone and major MGE interneuron subtypes, the direct lineage relationship between them remains to be experimentally verified. In mammals, it is well-established that MGE gives rise to progenitors that tangentially migrate and ultimately differentiate into PV + and SST + interneurons. However, in the absence of genetic fate mapping or time-lapse imaging, it remains a limitation of our hypothesis that the *nkx2.1*+/*lhx6a* + domain identified as teleost MGE is the main source of the *sst1.1* and *pvalb6* populations observed at later stages. A critical next step is to move from marker expression to definitive genetic lineage tracing. The development of tools such as Cre/loxP recombination systems driven by *nkx2.1* and *lhx6a* promoters could be used to label this progenitor pool and track cell differentiation. Lineage tracing studies utilizing transgenic lines for *dlx1a/2a* or *dlx5a/6a* suggest that these progenitors primarily give rise to GABAergic cells. However, these fluorescently labeled cells do not always colocalize with *HuC* or *gad65* expression and may only represent a sub-population of adult GABAergic interneurons (Solek et al., 2017). Future studies incorporating GE-specific promoters coupled to fluorescently labeled lineage-specific reporter lines with live light-sheet microscopy would enable direct visualization of their migration routes in real-time. This would resolve whether zebrafish interneurons undergo the tangential migration characterized by their mammalian counterparts, or if they employ distinct strategies to populate the mature teleost brain. Studies here establish a baseline for a better understanding of interneuron origins in zebrafish.

## Data Availability

The datasets presented in this article are not readily available due to privacy or ethical restrictions. Requests to access the datasets should be directed to the corresponding author.
