# Supplementary material for: Gene expression mapping of conserved GABAergic interneuron markers in developing zebrafish
Source: Front Mol Neurosci. 2026 Jul 2;19:1849805. doi: 10.3389/fnmol.2026.1849805 (PMC13373082; doi:10.3389/fnmol.2026.1849805)
Supplement: Supplementary file 1 [file Data_Sheet_1.PDF]

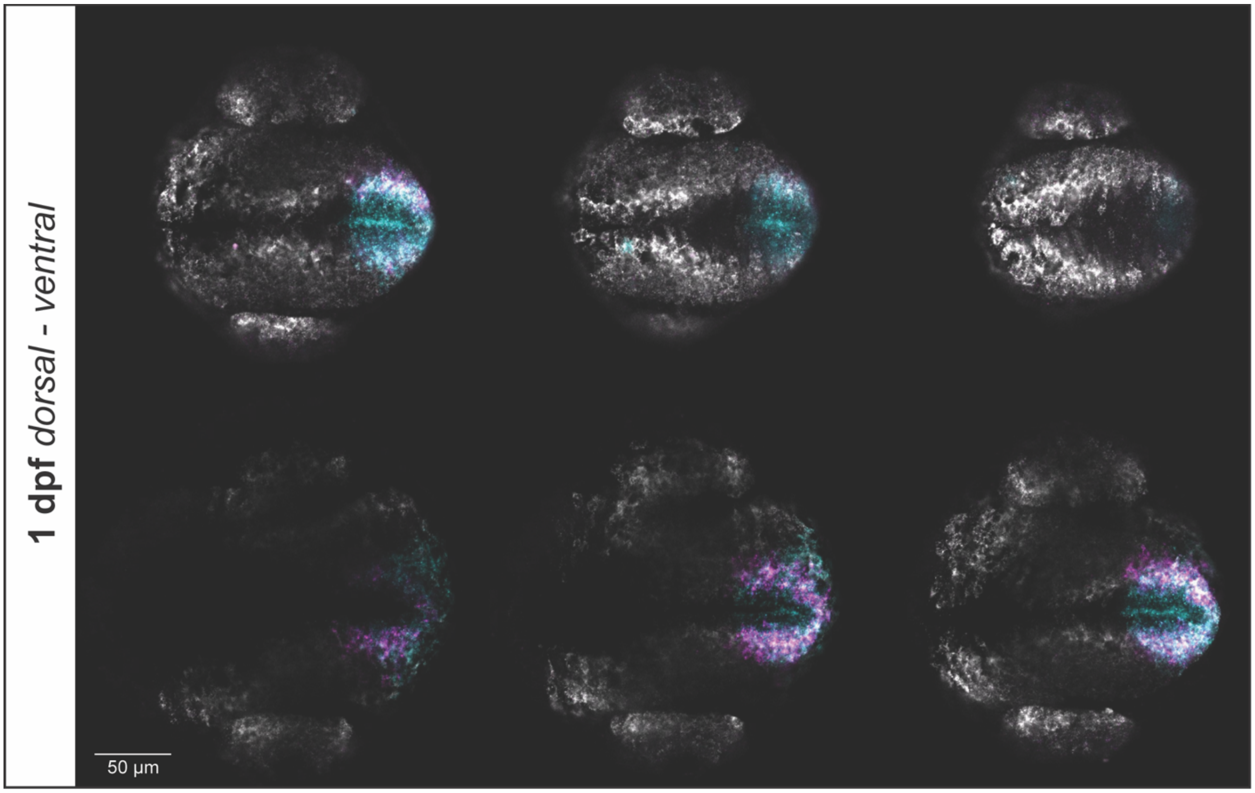

Supplemental Figure 2-1. Dorsoventral distribution of *nkx2.1* and *lhx6* expression at 1 dpf. Serial confocal optical sections of the forebrain arranged from dorsal to ventral planes. HCR staining for nkx2.1 (magenta) and lhx6 (cyan) reveals the volumetric extent of the subpallial progenitor domain. Huc (grey) serves as a pan neuronal counterstain. Scale bar = 50 µm. (n = 4). Abbreviations: OP, olfactory placode; Tel, Telencephalon.

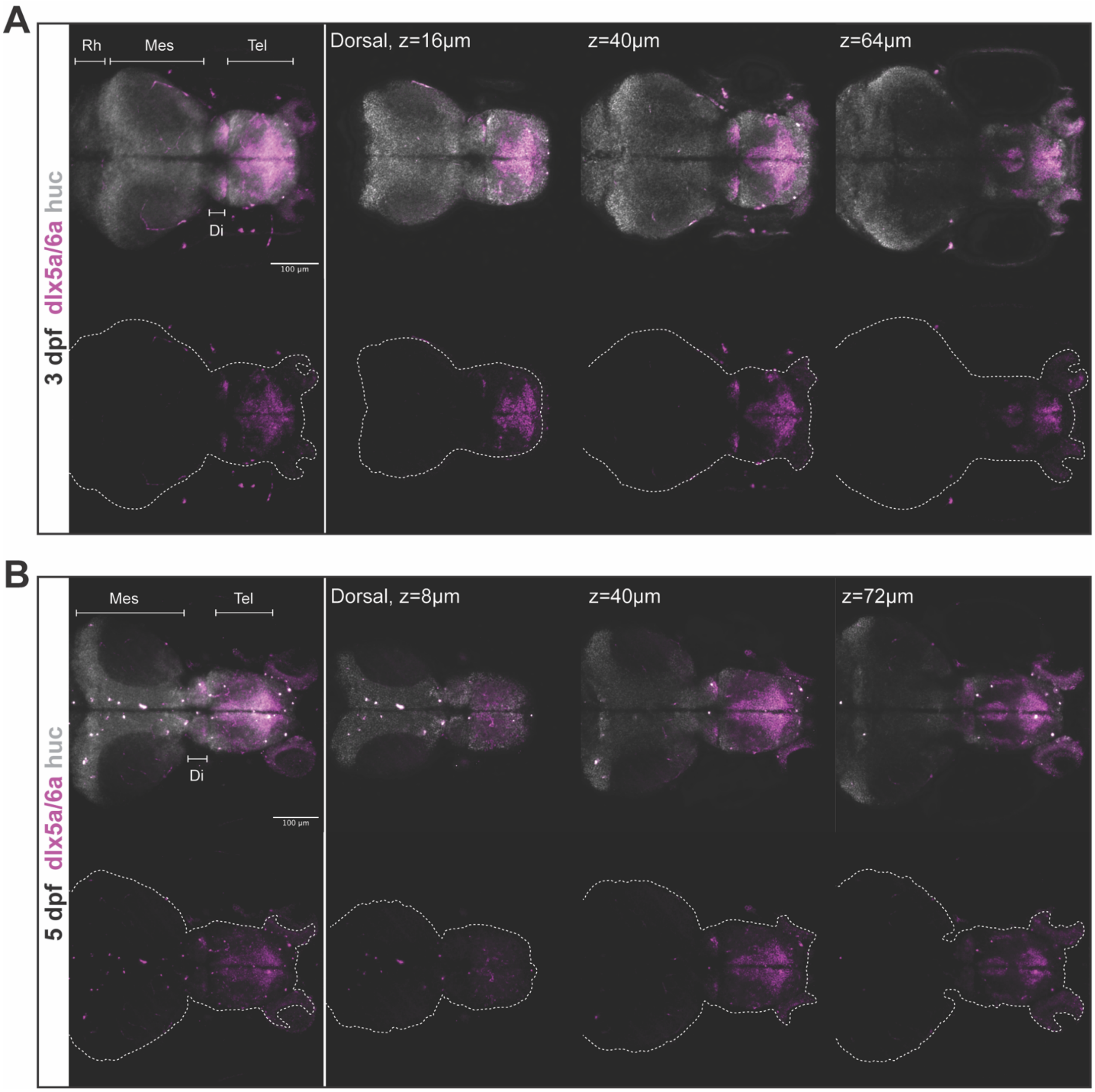

Supplemental Figure 3-1. Spatiotemporal distribution of *dlx5a/6a* expression at 3 and 5 dpf. A, B. Confocal analysis of interneuron *dlx5a/6a* (magenta) and pan-neuronal *huc* (grey, n = 5 larvae per condition). Gene markers are displayed as maximum intensity projection, while *huc* is displayed as an average intensity projection to visualize anatomical structures. Representative dorsal views (left) and corresponding serial optical sections (right) at 3 dpf (A) and 5dpf (B) reveal that *dlx5a/6a* expression is robustly maintained in the dorsal telencephalon and diencephalon through this developmental window. Scale bar = 100 µm. Abbreviations: Di, Diencephalon; Mes, Mesencephalon; Rh, Rhombencephalon; Tel, Telencephalon.

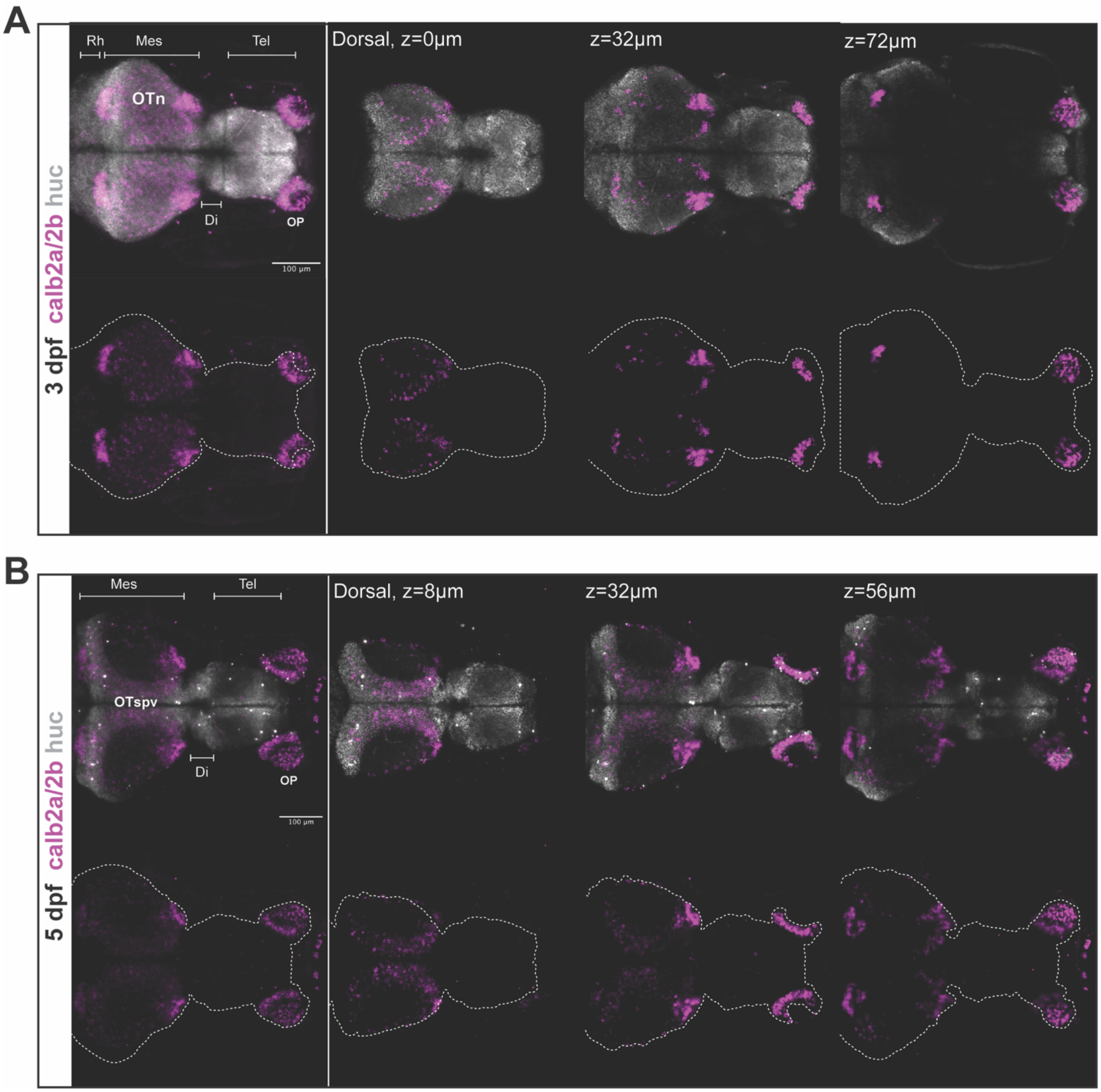

Supplemental Figure 4-1. Spatiotemporal distribution of *calb2a/2b* expression at 3 and 5 dpf. A, B. Confocal analysis of interneuron *calb2a/2b* (magenta) and pan-neuronal *huc* (grey, n = 5 larvae per condition). Gene markers are displayed as maximum intensity projection, while *huc* is displayed as an average intensity projection to visualize anatomical structures. At 3dpf, *calb2a/2b* is highly enriched in the olfactory placode, optic tectum and rhombencephalon (A), and maintained through 5 dpf (B). scale bar = 100 μm. Abbreviations: OP, olfactory placode; Di, Diencephalon; OTn, Optic Tectum; OTspv, Optic Tectum Stratum Periventriculare.

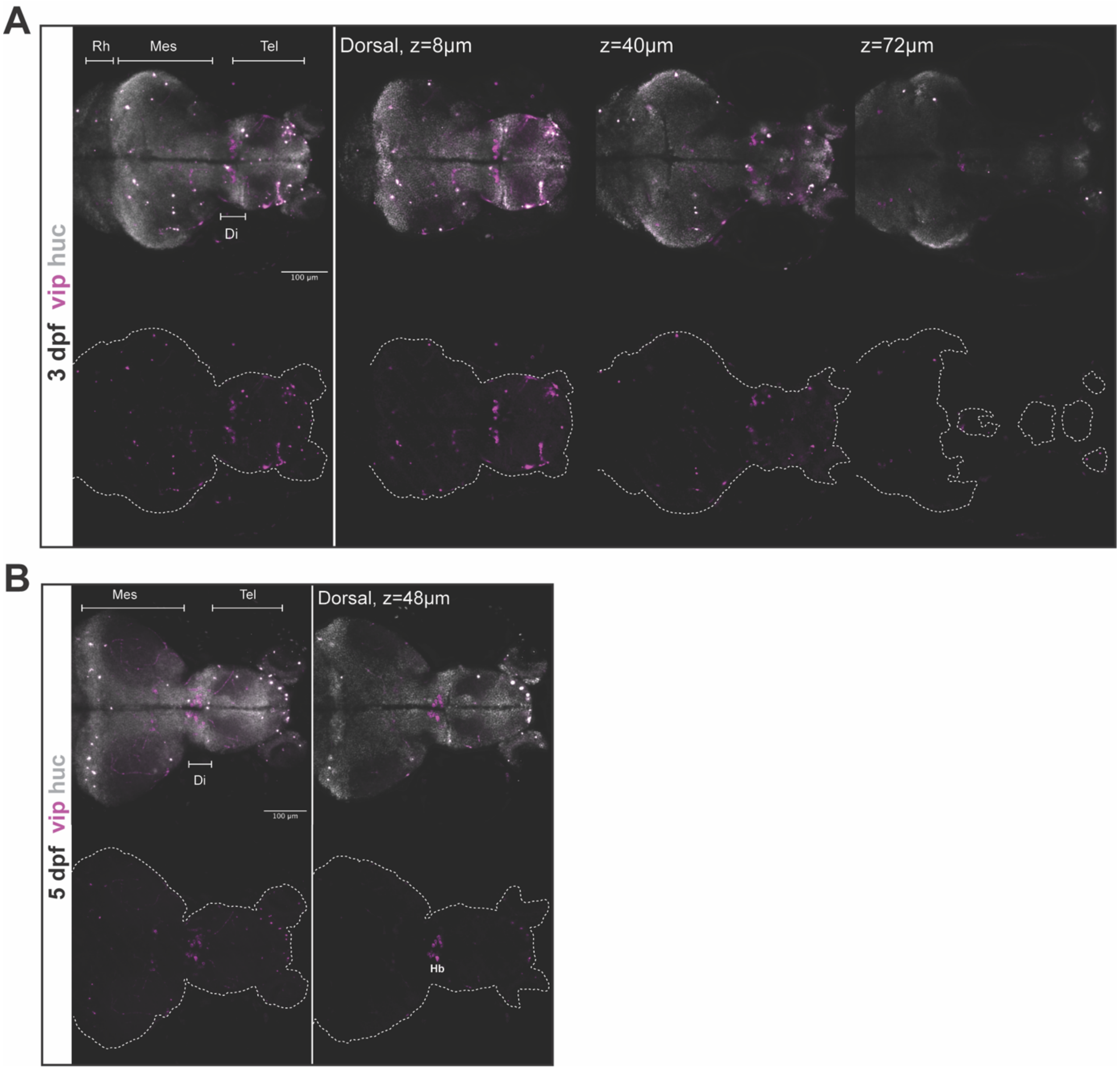

Supplemental Figure 5-1. Spatiotemporal distribution *vip* expression at 3 and 5 dpf. A, B. Confocal analysis of interneuron *vip* (magenta) and pan-neuronal *huc* (grey, n = 3-5 larvae per condition). Gene markers are displayed as maximum intensity projection, while *huc* is displayed as an average intensity projection to visualize anatomical structures. Representative dorsal views (left) and serial optical sections (right) at 3 dpf (A) and 5 dpf (B) reveal a highly restricted distribution pattern. Unlike other subtype markers, *vip* expression is limited to rare, solitary cells embedded primarily within the diencephalon, with minimal presence in the telencephalon or mesencephalon. Scale bar = 100 µm. Abbreviations: Rh, rhombencephalon; Mes, mesencephalon; Tel, telencephalon; Di, diencephalon; Hb, Habenula.
